# Rapid prototyping for high-pressure microfluidics

**DOI:** 10.1038/s41598-023-28495-2

**Published:** 2023-01-22

**Authors:** Carlie Rein, Mehmet Toner, Derin Sevenler

**Affiliations:** 1 Center for Engineering in Medicine and Surgery, Massachusetts General Hospital, Boston, MA USA; 2 Harvard Medical School, Boston, MA USA; 3 Shriners Hospital for Children, Boston, MA USA

**Keywords:** Biomedical engineering, Mechanical engineering, Lab-on-a-chip

## Abstract

Soft lithography has permitted rapid prototyping of precise microfluidic features by patterning a deformable elastomer such as polydimethylsiloxane (PDMS) with a photolithographically patterned mold. In microfluidics applications where the flexibility of PDMS is a drawback, a variety of more rigid materials have been proposed. Compared to alternatives, devices fabricated from epoxy and glass have superior mechanical performance, feature resolution, and solvent compatibility. Here we provide a detailed step-by-step method for fabricating rigid microfluidic devices from soft lithography patterned epoxy and glass. The bonding protocol was optimized yielding devices that withstand pressures exceeding 500 psi. Using this method, we demonstrate the use of rigid high aspect ratio spiral microchannels for high throughput cell focusing.

## Introduction

Rapid prototyping techniques accelerate early-stage development of microfluidic technologies by reducing iteration time and upfront costs. Perhaps the most widely used rapid prototyping technique for microfluidics research is soft lithography, which typically involves patterning a polydimethylsiloxane (PDMS) elastomer part from a mold of micropatterned photoresist film on a silicon wafer^1^. The relatively soft and tough PDMS part is lifted off from the rigid silicon mold. The major advantages of soft lithography, as compared with alternative rapid prototyping methods such as 3D printing, stem from the excellent feature resolution afforded by thin film photolithography on silicon wafers, as well as the ability to quickly produce multiple elastomeric devices from a single wafer mold.

However, the deformability of PDMS is unfavorable for microfluidics applications that involve moderate pressures and where the channel geometry is important. For example, this is the case for virtually all studies of inertial microfluidics phenomena, which generally involve relatively high-pressure flows (P > 30 psi) in relatively long microchannels (> 1 cm). PDMS devices start to deform at as little as 15 psi, and can rupture at pressures of about 40–60 psi^2^. Therefore, the use of PDMS can jeopardize both research results, where deformability can be a substantial source of experimental variability, as well as translational development, since scaled-up manufacturing processes overwhelmingly use thermoplastics which are much more rigid than PDMS. In these cases, it would be prudent to first validate microfluidic designs in a rigid material before bearing the substantial tooling costs for injection molding or embossing.

These considerations motivated the development of new techniques for rigid device prototyping by several groups, systematically reviewed in 2011^3^. These efforts demonstrated the fabrication of rigid devices from a photolithographically defined pattern by transfer molding using an intermediate PDMS replica. Among the materials evaluated, the thermoset recipe first described in 2007 exhibited the highest stiffness and best bonding performance (at least 150 psi)^4^. Later, another transparent thermoset, the epoxy resin EpoxAcast 690, was utilized to measure particle focusing at very high flow rates at operating pressures approaching 10,000 psi^5,6^. This same material was also shown to have excellent chemical inertness and gas impermeability^7^. It was also shown that an epoxy chip had the capability to capture circulating tumor cells from whole blood based on their size with an efficiency of about 80%^8^. Based on these studies, epoxy-glass devices may be considered to have equal or superior characteristics to all other studied rapid prototyping materials with regards to feature fidelity, rigidity, and bond strength. Altogether, compared to alternative rapid prototyping methods such as 3D printing or patterned laminate films, photolithographically patterned devices have the highest feature resolution and wall smoothness^9^. Likewise, commercially available epoxies are more accessible to researchers than custom materials^10^.

Despite the many advantages of rigid devices, rigid materials are rarely utilized in the literature even when warranted. A detailed step-by-step protocol may greatly improve the accessibility of rigid device prototyping to microfluidics laboratories that are already using soft lithography and PDMS. To address this need, we describe the most common pitfalls in mold preparation and device bonding, how they may be addressed. Furthermore, we optimize several key process parameters to maximize bond strength while maintaining the ease and limited resources of the protocol.

## Results

### Overview and key points of protocol

**Figure 1:**
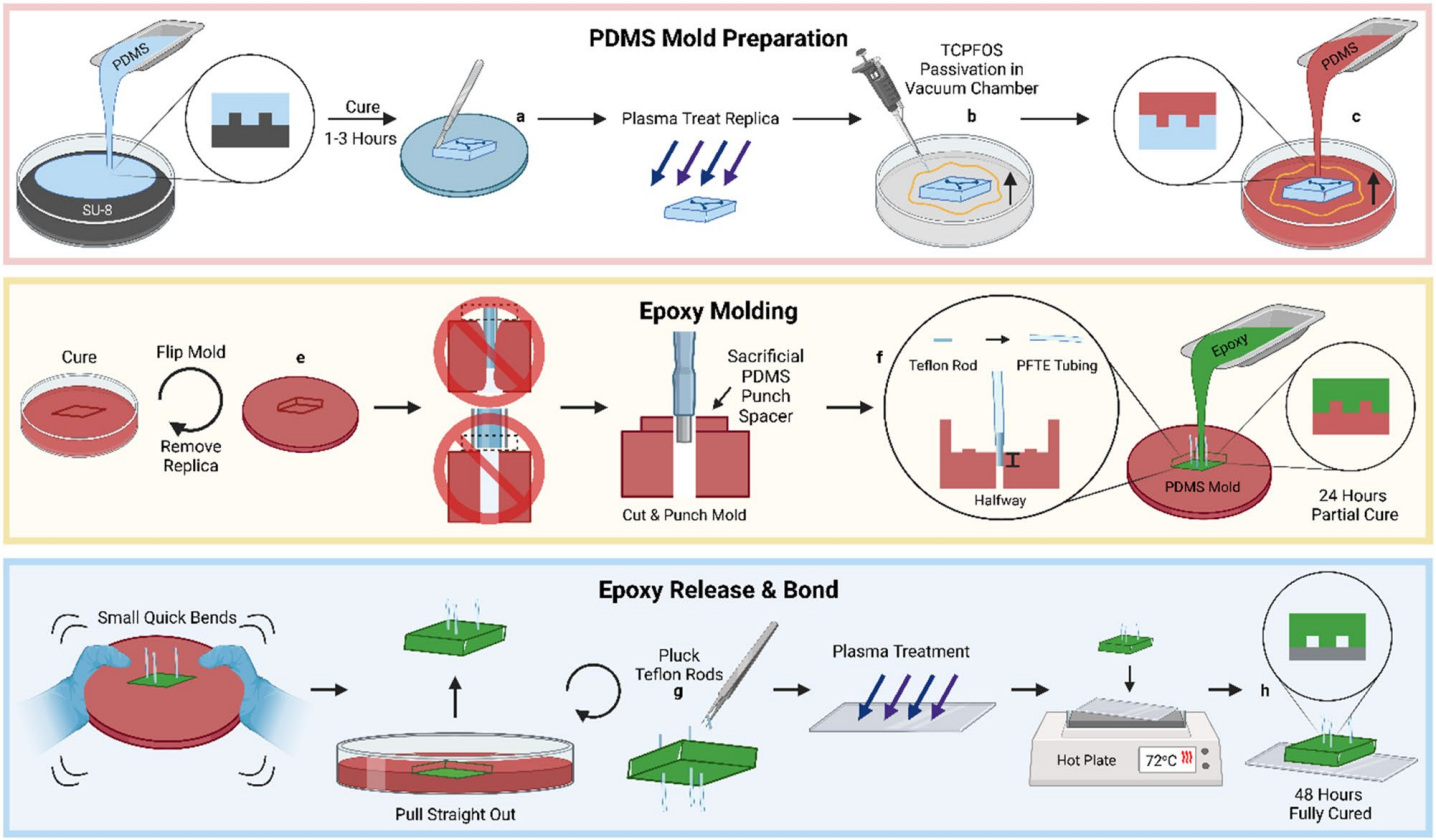
Overall procedure to fabricate epoxy-based microfluidic device.

**Figure 2:**
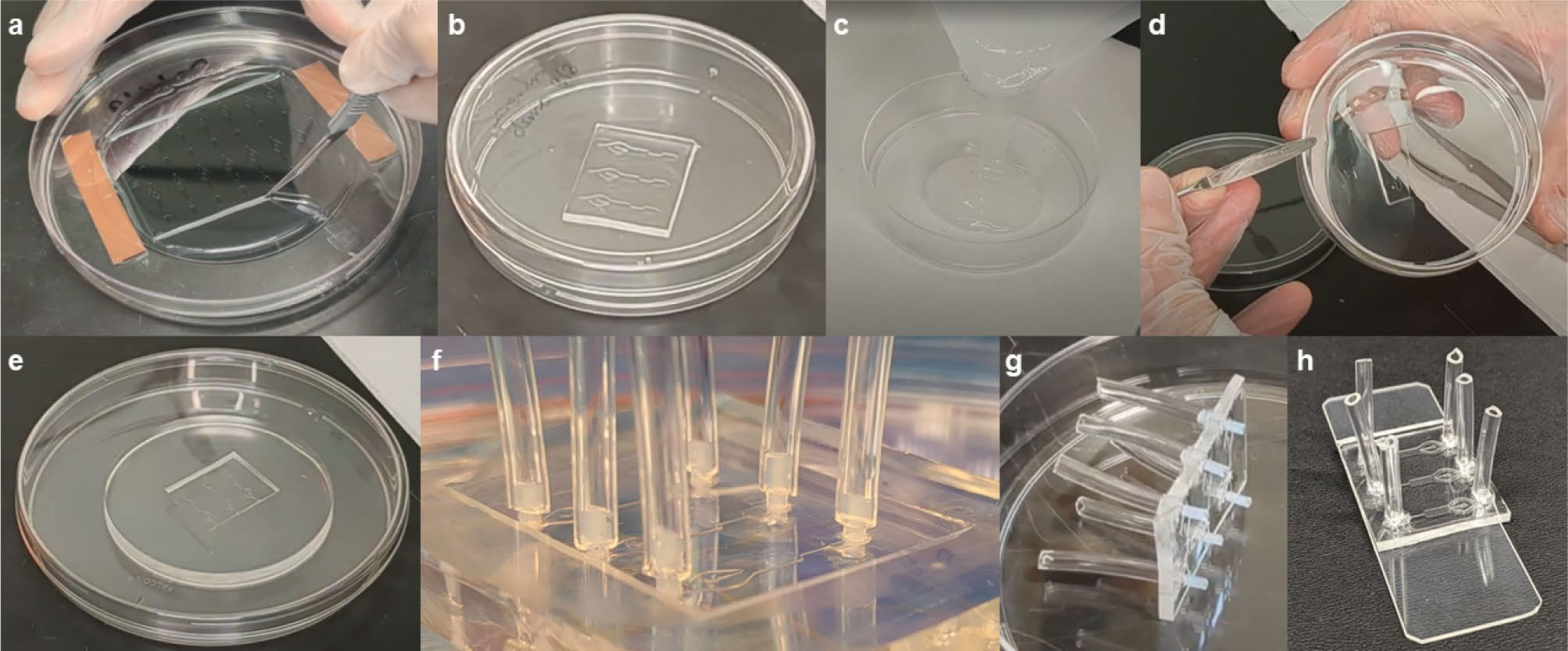
Example images of protocol stages. From left to right and top to bottom: (**a**) cutting PDMS from SU-8 wafer, (**b**) PDMS replica in the petri dish facing up, (**c**) pouring PDMS over replica, (**d**) releasing the PDMS mold from the replica, (**e**) the final PDMS mold without the replica device, (**f**) PDMS mold with tubing with no epoxy, (**g**) epoxy device taken out of mold after 24 h with PFTE rods still attached sitting on its side, and (**h**) the final epoxy device bonded to a glass slide.

The procedure consists of three main steps: (1) PDMS mold preparation, (2) epoxy pouring, and (3) mold release, post-processing, and bonding (schematic, Fig. 1 and example images, Fig. 2). After mold preparation (1), the mold can be stored securely, and the rest of the fabrication can be conducted at any time. When the epoxy is poured (2), the mold release (3) is conducted on the following day, after 20–24 h, and bonding is conducted immediately after mold release (3). This procedure assumes you have familiarity with conventional soft lithography of PDMS using a rigid silicon wafer mold^1^. Before you begin, you will need a master mold with the desired patterned features, such as a patterned SU-8 thin film on a silicon wafer. As described below, the *channels* in the final device should be *raised features* on the master mold, just like for conventional PDMS devices.

Regarding the protocol as a whole, we had several important observations. First, the TCPFOS-treated replicas which are used to make the PDMS mold can be reused, but only 2–3 times. Beyond that, the surface of the replica can become warped. On the other hand, the PDMS device molds can be reused many (10+) times. Over time, the mold can acquire an opaque white color. It does not affect the device or the PDMS mold. Furthermore, cutting the Polytetrafluoroethylene (PFTE) rods at a slight angle can make it easier to insert them into the molds. Finally, the tubing material should be selected to be compatible with epoxy bonding. We use polyethylene tubing, which forms a strong bond with the epoxy. Common tubing materials such as Tygon^®^ or Fluorinated ethylene propylene (FEP) will not react with the epoxy.

### Optimization of epoxy fabrication

We optimized several process variables with the goal of being able to reliably make devices that could withstand high pressures. Cure time was optimized before mold removal, the plasma treatment time, and the duration of resting time on the hot plate after bonding. To test the bond strength of a device, we fabricated the device with just a single port (i.e. no outlets) and connected it to the high-pressure syringe pump model 100Dx with an integrated pressure transducer. The device was gradually pressurized with DI water over the course of several minutes until failure, and the peak pressure before failure was recorded.

**Figure 3:**
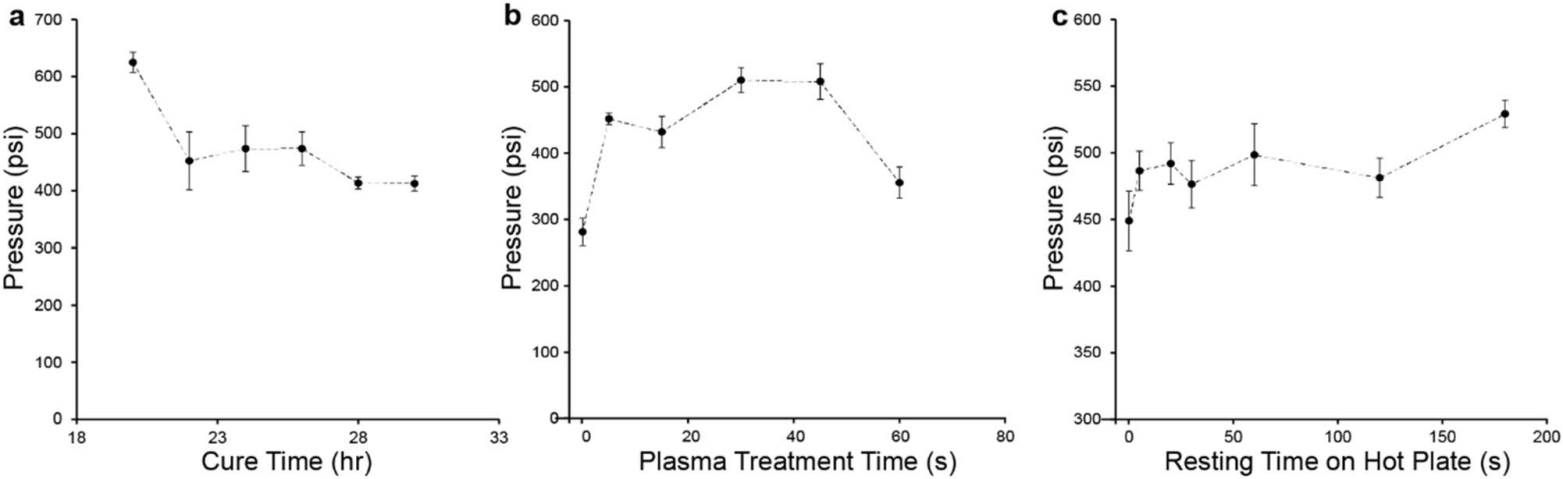
Optimization of bond strength. Bond strength versus (**a**) cure time, (**b**) duration of plasma treatment, and (**c**) time the device rests on a hot plate after plasma treatment. Pressures range from 650 to 240 psi. Strength was tested by connecting a high-pressure pump to the devices using a Swagelok with an initial flow rate of 1 ml/min and final flow rate of 0.1 ml/min. The final average weight of all devices tested is 6.57 g.

First, we hypothesized that using a shorter cure time than 24 h may increase the bond strength if the epoxy has more available reactive groups. For cure times less than 20 h, the epoxy was too soft and tacky to remove from the mold. On the other hand, for cure times over 30 h, we observed that the epoxy had almost fully hardened and would not bond to the glass slide. Within this window, we observed a trend towards higher bonding pressures at lower cure times, with a maximum of 670 psi at the lowest tested cure time of 20 h (Fig. 3a).

Next, we evaluated the effects of plasma and hot plate time on bond strength. Plasma treatment of the glass slide cleans the surface and makes it more hydrophilic. Based on the manufacturer of the plasma generator with an advised starting point of 20 s, the range of plasma treatment times tested spanned from zero to 60 s. All devices that received at least 5 s of plasma treatment performed similarly (Fig. 3b) with the highest pressure reaching an average of 510 psi after 30 s. The devices that received no plasma treatment had the lowest bonding strength, failing at roughly half of the pressure plasma-treated devices were able to reach. The range of hot plate times tested spanned from 0 to 3 min. At 3 min on the hot plate, devices reached its average maximum at 530 psi before failing. After 3 min, however, the devices were observed to sometimes warp and lift away from the glass surface. Nevertheless, devices that received more heat also seemed to be stronger (Fig. 3c). Resting times on the hot plate under 1 min showed no differences.

Since the pressure sweep experiments were relatively rapid (~ 2 min), we also tested whether devices would withstand similarly high pressures for a prolonged period. We fabricated a device using the protocol described above at a 24-h cure, and pressurized at increments of 100 psi, increasing by 100 psi every 10 min. We observed that the device was able to withstand a pressure of 100 psi for an hour. It was also able to last for at least 10 min at 200 and 300 psi. The device failed at 400 psi after 6 min, which was similar to the device strength expected based on the pressure sweep experiments (Fig. 3b).

Finally, we fabricated two final chips using a protocol which, based on the optimization results, was expected to be optimal for maximizing bond strength (a 20-h cure time, 30 s for plasma treatment, and 3 min on the hot plate). This resulted in a device that could withstand an average of 714.5 psi based on two chips, about 12% higher than the prior highest condition during optimization.

### Inertio-elastic cell focusing in a rigid high aspect ratio spiral microchannel

We used the optimized fabrication protocol to make spiral microchannels with a very high aspect ratio and test their performance for high throughput inertio-elastic cell focusing. For biomedical applications of inertial focusing involving large sample volumes, the maximum shear stress on the cells must remain within limits to avoid damage. For reasons discussed later, we hypothesized that a very high aspect ratio microchannel (i.e. 10:1 or more) would have several advantages. However, high aspect ratio channels are also more prone to inflating under pressure if they are fabricated from deformable materials.

**Figure 4:**
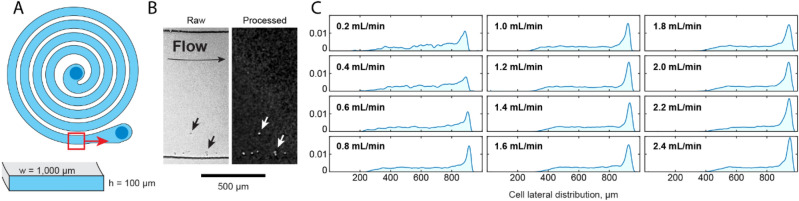
Inertio-elastic cell focusing in a rigid and high aspect ratio spiral microchannel. (**a**) Schematic of microchannel geometry Inlet is at center of spiral. (**b**) Representative raw and processed images of Jurkat cells under flow in a viscoelastic suspension of hyaluronic acid. (**c**) Focusing performance quantified by lateral distributions of cells from inner (convex) to outer (concave) wall, at increasing flow rates.

We fabricated a single microchannel 100 µm tall, 1 mm wide, and about 140 mm long, coiled into a spiral about 17 mm wide altogether (Fig. 4a). A viscoelastic solution of hyaluronic acid was prepared by dissolving 1.5 MDa lyophilized HA in phosphate buffered saline to a concentration 0.5 mg/ml by gentle mixing overnight. Jurkat cells about 12 µm were resuspended in the viscoelastic solution and pumped into the center of the spiral at flow rates up to 2.4 ml/min. Cells within the channel were imaged with 10× brightfield microscope (Fig. 4b). The lateral distribution of cells near the outlet was quantitated by acquiring > 100 timelapse images at each flow rate, followed by image processing to segment cells and accumulate their lateral positions (Fig. 4c). As expected, above a critical flow rate of about 0.8 ml/min, a plurality cells were focused to a single stable point close to the outer (i.e. concave) wall of the microchannel. Furthermore, the apparent stable point moved closer to the outer wall with increasing flow rate, consistent with expectations. Above 1 ml/min, focusing characteristics continued to slightly improve with increasing flow rate, through the highest tested flow rate tested.

## Conclusion and discussion

Epoxy-based microfluidic devices resist deformation and withstand higher pressures than PDMS devices. In developing this protocol, we build upon prior work identifying epoxy as a high performance material, with the goal of making this protocol more accessible to microfluidics laboratories around the world who are already using PDMS and soft lithography^5–7^.

Using a rigid epoxy device, we studied the focusing performance in high aspect ratio microchannels. Compared with moderate aspect ratio microchannels (i.e., 4:1 or less), high aspect ratio curved microchannels have the advantage of suppressing the onset of Dean flow secondary vortices at higher Reynolds numbers, which permits efficient cell focusing at higher flow rates^11^. Based on the measurements of similar compounds by others, the fluid mechanical properties of the solution (1.5 MDa, 0.5 mg/ml hyaluronic acid in PBS) may be roughly estimated as a zero-shear viscosity of about 5 mPa s, and a relaxation time of about 10 ms. With a characteristic channel width of 100 µm, the highest tested flow rate of 2.4 ml/min would correspond to a Reynolds number of about 8 and to a Weissenberg number of about 40. The observed focusing characteristics are consistent with results by others investigating flows with a similar Elasticity Number, here El = Wi/Re = 4.8^12,13^.

This protocol has several potential limitations. One potential limitation of this procedure is that it is not ideal for high aspect ratio features such as pillars or thin walls because the mold is released when the epoxy part is still pliable. Releasing and bonding devices with very high aspect ratio features may take additional optimization and practice. Nevertheless, epoxy provides excellent mechanical properties and surface finish for high throughput applications. This detailed protocol will allow more laboratories to easily integrate rigid devices into their existing PDMS-based prototyping workflows.

## Materials reagents and equipment

### Reagents

- The pattern master mold, e.g. a micropatterned SU-8 thin film silicon wafer. *Channels* in the final device should be *raised features* on the master mold, just like when using it to mold a conventional PDMS device.
*EpoxAcastTM 690 transparent epoxy kit (https://shop.smooth-on.com/epoxacast-690).
*Trichloro(1H,1H,2H,2H-perfluorooctyl)silane (TCPFOS) (Millipore Sigma, Item No. 448931, https://www.sigmaaldrich.com/US/en/product/aldrich/448931).
*Sylgard 184 silicone elastomer kit (Ellsworth Adhesives, Item No. 4019862, https://www.ellsworth.com/products/by-market/consumer-products/encapsulants/silicone/dow-sylgard-184-silicone-encapsulant-clear-0.5-kg-kit/).

### Materials

- Polytetrafluoroethylene (PFTE) Rod 0.07″ (https://www.mcmaster.com/84935K82/).
- Polyethylene (PE) tubing 1/16″ ID (https://www.mcmaster.com/5648K67-5648K226/).

### Equipment

- Plastic Petri dishes 100 mm.
- Petri Dishes 175 mm with lid.
- Weigh boat.
- Hot plate.
- Ultraclean glass slides (Fisher Scientific, Item No. 22-037-213, https://www.fishersci.com/shop/products/ultraclean-microarray-slides-enhanced-surface/22037213?searchHijack=true&searchTerm=+C22-5128-M20&searchType=RAPID&matchedCatNo=+C22-5128-M20).
- Clean metal spatula with rounded end, e.g. (https://us.vwr.com/store/catalog/product.jsp?product_id=4531745).
- Pipettes.
- Separate Vacuum chamber for TCPFOS vapor deposition (https://www.fishersci.com/shop/products/bel-art-scienceware-space-saver-vacuum-desiccators-3/0859416A?gclid=CjwKCAiAvK2bBhB8EiwAZUbP1OvPjQ-ZJAhbEmrVHglNDxRCoS1kEZrG47Yro7f3h4sNqjjlgiL0dRoCh7YQAvD_BwE&ef_id=CjwKCAiAvK2bBhB8EiwAZUbP1OvPjQ-ZJAhbEmrVHglNDxRCoS1kEZrG47Yro7f3h4sNqjjlgiL0dRoCh7YQAvD_BwE:G:s&ppc_id=PLA_goog_2086145680_81843405274_0859416A__386247001354_9967139677844655410&ev_chn=shop&s_kwcid=AL!4428!3!386247001354!!!g!827721591040!0859416A).
- Toothed Forceps, also called ‘tissue’ or ‘rat tooth’ forceps, e.g. (https://www.wpiinc.com/14140-g-graefe-forceps-7cm-straight-07mm-1x2-teeth-german ).
*Plasma Generator (https://www.enerconind.com/plasma-treating/products/vintage-treaters/dyne-a-mite-hp.aspx).
- Electric Ultra-High-Vacuum Pump (Item No. 4396K21, https://www.mcmaster.com/catalog/128/421).

*Be sure to read over the Safety Data Sheets for these products and wear appropriate PPE while using them.

## Procedure

The protocol is ideally performed in a clean room environment to avoid particulate contamination.

### PDMS mold preparation

In this step, we will first prepare a PDMS *replica* from the silicon wafer master mold, then use it to cast a PDMS *mold*. The PDMS mold will need to be punched to allow inlet and outlet tubing to be held in place during the epoxy casting step below. Likewise, the PDMS master molds can be reused to cast epoxy devices many (10+) times.

This protocol is for making devices sized similar to or smaller than a standard glass slide. For small devices (e.g. 1–3 cm^2^) it is possible to cast a single PDMS mold from multiple PDMS replicas.

One potential pitfall of mold preparation is to make the punched holes either slightly too large or too small. In a later step, PFTE rods will be inserted into the holes. The holes should be just slightly undersized (~ 5%) from the PFTE rod diameter, to allow a tight press to fit and prevent epoxy from seeping through. On the other hand, if the punched holes are too undersized, it may still be possible to fit the rod inside, however this will stretch the PDMS surface and cause it to pull down, by virtue of the Poisson effect. This pulldown will then be carried over to the epoxy part and may cause issues with bonding.

A second potential pitfall with mold preparation, also associated with hole punching, is hole shape. A conventional round biopsy punch can compress a PDMS part substantially before biting through, causing a necking-down of the hole with depth in the relaxed PDMS part. This hole shape can leave a small ‘countersunk’ perimeter around a press-fit PFTE rod, and this feature can be carried over to the epoxy part and cause issues with bonding.

To avoid these two issues, we use a 2 mm thick sacrificial PDMS spacer on top of the PDMS mold during punching. In our experience, a 2.0 mm biopsy punch can be used to make straight-walled holes 1.8 mm in diameter, which are suitable for 0.07″ PFTE rod stock and 1/16″ inner diameter tubing.

### PDMS pouring

*Time: 15 min*

1. Make a PDMS mixture total of 50 g (45 g of PDMS Base; 5 g of Curing agent). Mix well.
2. Pour the PDMS over the SU-8 wafer.
3. Place the dish with the SU-8 wafer and PDMS into vacuum chamber. Leave the dish for 1–3 h, then transfer to a 60 °C oven to cure the PDMS overnight.

### Release, cut device replicas

*Time: 15 min*

4. Gently remove the PDMS casting from the silicon mold.
5. Cut out the replica to size from the PDMS.

### Replica surface passivation

*Time: 35 min*

6. Perform oxygen plasma treatment of the PDMS replica.
7. Place replica in a clean 100 mm petri dish.
  NOTE: If the devices are small, there may be space to fit multiple replicas in a single 100 mm petri dish mold. Make sure the replicas do not touch each other in the dish, ideally at least 5 mm of space between replicas as well as between any replicas and the petri dish wall.
8. Pipette and lightly spread 2 μl of Tricholorperfluorooctylsilane (TCPFOS) onto the petri dish and to make a border around the replicas.
  NOTE: TCPFOS is toxic. Use appropriate PPE.
9. Place the petri dish into a dedicated vacuum chamber for 30 min.
  NOTE: Small amounts of TCPFOS may be deposited on both the inner and outer surfaces of the petri dish and should be handled accordingly following appropriate safety precautions. Do not handle the dish without gloves.

### Pour PDMS onto replicas

*Time: 15 min*

10. Make a PDMS mixture total of 50 g (this will give ~ 10 mm thick PDMS in a 100 mm petri dish, e.g. for casting on replicas which are ~ 5 mm thick; 45 g of PDMS Base; 5 g of Curing agent). Mix well.
11. Pour the mixture into the 100 mm dish with the devices. Be sure they are fully covered.
  NOTE: Make note of how many times this replica has been used. Do not got past 2 uses. Future devices will become warped.
12. Place the dish into vacuum chamber. Let the dish sit for 1–3 h, then transfer to s 60 °C oven to cure PDMS overnight.
  OVERNIGHT.

### Release and punch PDMS mold

*Time: 15 min*

#### Release

13. Using a metal spatula, gently remove the PDMS mold completely out of the petri dish.
  NOTE: It may help to squeeze opposite sidewalls of the plastic petri dish very gently towards one another, to slightly flex the entire dish and allow the PDMS to separate from the dish. The replica may start to fall out of the mold during this step. Take care not to damage or tear the mold or the replica at this step.
14. Using a clean metal spatula or tweezers, gently remove the PDMS replica from the mold.
  NOTE: It may help to gently flex the entire PDMS mold until the replica begins to separate from the mold. Be sure not to damage or tear the mold or the replica at this step.
15. Cut off the extra lip from the edge of the PDMS mold. Test the level of it on the table and lightly tap; it should be flush with the table.

#### Punch

16. Using a 2.0 mm hole punch, punch out the holes necessary. Lift the PDMS and punch out the PDMS plug from the other side.
  NOTE: We recommend using a spare piece of PDMS on top of the hole being punched. This prevents the necking-down of the hole described in “PDMS mold preparation” section of the protocol.

### Prepare tubing inserts

As noted in the key points, certain materials like Tygon^®^ and FEP do not react with epoxy. If other materials outside this protocol are used, make sure they react with epoxy.

The purpose of inserting the PFTE rod into the PE tubing is to hold the PE tubing in place during the epoxy pouring and curing. It also ensures an open connection between the tubing and the microfeatures in case any epoxy leaks into the hole. In “Release and bond” section of the protocol, the rods are later on taken out from underneath the device.

Be sure to clean the surface being used and the tubing with 70% ethanol before starting.

*Time: 5 min*

#### Preparation

17. Cut the PFTE rod to 6 mm and the PE tubing to 2 cm.
  NOTE: Cutting the PFTE tubing at an angle can help guide the tubing during insertion.
18. Place the PFTE rod into the PE tubing. The rod does not need to go all the way into the larger tube; 1 mm deep is adequate.

#### Insertion

19. Place the tubing, rod first, into the holes of the mold in one smooth motion. The tubing should be at least 1–2 mm below the top of the PDMS mold. Do not push the tubing all the way down through the mold. Halfway down or less into the mold is sufficient; the tubing should have about 1–2 mm of space above the mold. See Fig. 1 for reference. Take care not to damage or tear the mold surface during this step.
  NOTE: You may leave the petri dish with mold and tubing in a vacuum chamber for about 15 minutes while preparing the epoxy mixture below. In our experience, degassing the PDMS mold before pouring the epoxy may help prevent small bubbles in the final epoxy part.

## Epoxy

All epoxy work should take place in a well-ventilated area. Read over all safety data sheets before using epoxy. The epoxy used in this protocol has a potting life of 5 h. Epoxy spills can be cleaned with 70% ethanol. If a micropipette is used to pour the epoxy for smaller molds, be sure to use tips with filters to avoid epoxy entering into the micropipette.

### Pour epoxy

*Time: 10 min*

#### Mix epoxy parts

20. Measure out the required amount of mixture into a weigh boat. Shake Epoxy mixture bottles well before use. The ratio of Part A to Part B to use is 10 g:3 g.
  NOTE: The densities of both Parts are different from water. The mass of added parts must be measured directly. Part A is much more viscous than Part B. One strategy for accurate preparation is to pour an approximate amount of Part A directly from the bottle, then calculate the precise amount of Part B to match the 10:3 ratio and add Part B gradually.
21. Bring the mixture into a fume hood. Mix very well for 3 min.
22. Place the epoxy mixture into the electric ultra-high-vacuum pump to degas the epoxy.
23. Watch for air bubbles forming. Bubbles will rise to the surface, combine, and then pop. Turn off machine when the first bubble begins to boil.
  TROUBLESHOOT: If there are still many bubbles leftover after turning the pump off, let the bubbles sit and pop by themselves for a few minutes before releasing the air. If there are still bubbles in the epoxy after degassing, run the vacuum again.

#### Epoxy pouring

24. Aliquot the epoxy mixture very slowly to the lowest point of the mold. Let the epoxy spread into the mold on its own. Do not let the epoxy overflow or bubble over. It should be leveled with the top of the PDMS mold.
  NOTE: If the mold bubbles over, you can use a pipette to remove excess epoxy. A Kimwipe^®^ can also be used to absorb excess epoxy by touching the top of the epoxy mold until it becomes level.
  TROUBLESHOOT: Small air bubbles trapped on the PDMS features may be visible. It may help to use a plastic 10 μl pipette tip and gently brush the mold to release bubbles from the mold surface and let them rise to the top of the epoxy. Any bubbles floating on top of the epoxy are acceptable and usually burst during the cure.
25. Place the petri dish in the fume hood. Leave any excess epoxy in the fume hood for 24 h before discarding it.
26. Leave the epoxy to cure for overnight, typically about 22–24 h depending on ambient temperature and epoxy thickness. As we describe above, devices cured for longer times are generally easier to release from the mold but have lower bond strength.
  *OVERNIGHT*

## Release and bond

Variability in bonding ability is dependent on this section of the procedure. These effects from cure times, plasma treatment time, and resting time on the hot plate are all observed and described in the results. Pay attention to small details such as “feathering”, extra epoxy still attached to the device that may have emerged from plucking out the rod, occurring after the removal of the PFTE rod. Full bonding could be affected by tampering with the bottom of the device after removal from the PDMS mold. Bonding devices with thin walls and other delicate features will require practice.

When using the plasma generator, take appropriate safety precautions.

*Time: 10 min*

### Release

27. Perform quick little bends around the edges of the devices. Partially-cured epoxy should feel firm, rubbery, and tacky. It is visible from under the device if the edges are releasing from the PDMS.
  TROUBLESHOOT: If the devices are not clearly releasing from the mold at this stage, they are too soft and require more time to cure. Check back in 1 to 2 h and try again.
28. Holding onto the tubes, quickly pull the devices straight out of the PDMS. The epoxy part may deform during mold removal, but it will begin to relax back to its molded shape within a few seconds after release.
  NOTE: Do not turn the rod. With your other hand, you can also try bending the mold back away from the epoxy device. This helps to partially release the device and pull the PDMS walls away as you pull the device out.
29. Holding onto the corresponding tube, pluck out the PFTE rods very quickly from underneath using tweezers. Do not touch the bottom of the chip. Adding a small bend to the tube helps, though do not twist the tube. Leave the device in dish on its side once the pieces are out.
  NOTE: Going slowly can leave a bend in the chip. It is recommended to use the tweezers shown in the “Equipment” section, as they grip the rod easily.
  TROUBLESHOOT: For any leftover “feathering”, use the tube recently removed to gently push the feathering back into the hole.

### Bond

30. Bring the hot place to about 70 °C.
31. Perform the oxygen plasma treatment of a clean glass slide.
  NOTE: Plasma treated glass gradually loses its reactivity in atmosphere and should be used as quickly as possible, ideally within minutes.
32. Move the glass slide to the hot plate.
  NOTE: Avoid touching the surface to be bonded.
33. Gently drop the device onto the glass slide. Leave on the hot plate for 20–30 s. Tap the top of the epoxy lightly to ensure the device is on the glass and there are no air bubbles. If pressed too hard, channels can collapse. Bonded parts of the device are visible from above.
34. Using a light source behind you, check in the reflection of the glass for air bubbles. Parts that are bonded to the glass are darker than the unbonded areas. Most can be taken out by pressing the chip firmly into the glass slide directly on top of the bubble. Beware that thin walls and channels can also easily collapse here if the device is pressed too hard.
  NOTE: This step is more challenging for high aspect ratio channels and may require practice.
35. Let the new epoxy-made microfluidic device sit for 24 h to fully cure before using it for any experiments. Devices can be stored at an ambient temperature while it finishes curing and after completing the full procedure.

## Data Availability

The data collected from this study are available from the corresponding author on reasonable request.
